# Large-Scale Modeling of Epileptic Seizures: Scaling Properties of Two Parallel Neuronal Network Simulation Algorithms

**DOI:** 10.1155/2013/182145

**Published:** 2013-12-15

**Authors:** Lorenzo L. Pesce, Hyong C. Lee, Mark Hereld, Sid Visser, Rick L. Stevens, Albert Wildeman, Wim van Drongelen

**Affiliations:** ^1^Department of Pediatrics, The University of Chicago, Chicago, IL 60637, USA; ^2^Computation Institute, The University of Chicago and Argonne National Laboratories, Argonne, IL 60439, USA; ^3^Mathematics and Computer Science Division, Argonne National Laboratories, IL 60439, USA; ^4^Department of Neurology, The University of Chicago, Chicago, IL 60637, USA; ^5^Committee on Computational Neuroscience, The University of Chicago, Chicago, IL 60637, USA

## Abstract

Our limited understanding of the relationship between the behavior of individual neurons and large neuronal networks is an important limitation in current epilepsy research and may be one of the main causes of our inadequate ability to treat it. Addressing this problem directly via experiments is impossibly complex; thus, we have been developing and studying medium-large-scale simulations of detailed neuronal networks to guide us. Flexibility in the connection schemas and a complete description of the cortical tissue seem necessary for this purpose. In this paper we examine some of the basic issues encountered in these multiscale simulations. We have determined the detailed behavior of two such simulators on parallel computer systems. The observed memory and computation-time scaling behavior for a distributed memory implementation were very good over the range studied, both in terms of network sizes (2,000 to 400,000 neurons) and processor pool sizes (1 to 256 processors). Our simulations required between a few megabytes and about 150 gigabytes of RAM and lasted between a few minutes and about a week, well within the capability of most multinode clusters. Therefore, simulations of epileptic seizures on networks with millions of cells should be feasible on current supercomputers.

## 1. Introduction

Biological systems are complex and networks of neurons are no exception. Simulating these systems provides a means for testing configurations that would be difficult or impractical to replicate in vitro or in vivo. Taking a computational approach can also enable sweeps in configuration space that would otherwise be intractable, as it often requires extremely large sample sizes to achieve any degree of significance because of the inherently low power of multidimensional exploratory data analysis [1, 2]. One approach to study how the many possible combinations of parameter values measured can affect experimental findings across scales is via modeling and large-scale simulations [3–17]. Furthermore, the behavior of macroscopic neural tissues depends on factors determined across a range of physical scales, from microscale (a few to a hundred neurons) to the meso- and macroscales (the emergent behavior produced by the simultaneous interaction of millions of neurons). Science has dramatically improved our ability to study the micro- and macroscales independently, but to date only simulations are capable of trying to infer the emergent macroscopic behavior from microscopic properties, even though more sophisticated tools are being developed [18, 19]. In fact, the problematic gap between scales in neuroscience has even reached the non-scientific press [20].

Simulations of these large neuronal populations require parallel computing, spreading the work across many processing units, in order to reduce the execution time into a practical regime [7, 15, 21, 22]. However, it is rarely easy to design an algorithm that will take half the time when twice the processor count is used for the computation, particularly over a wide range of these doublings. Scalability, the measure of an algorithm's ability to do this, is characterized in two distinct ways referred to as strong and weak scaling [23]. Strong scaling refers to the time to completion of an algorithm applied to a fixed-size problem as a function of the number of allocated processing units. It is sometimes characterized by a quantity called speedup, the ratio of the wall clock time (the time interval measured on the wall clock in the office of a scientist waiting for a calculation to complete) necessary to complete a task on a small number (usually 1) of processors (or nodes) and the time necessary to compute it with many more processors. In the case of linear speedup, the problem is solved *n* times faster if *n* times as many processors are allocated to work on it in parallel. Increasing the number of processors will not necessarily result in linear speedup because communication, the key expense incurred by parallelism, of interactions in simulated networks (e.g., spikes) might eventually dominate execution time. Weak scaling refers to the behavior of an algorithm applied to a problem whose size increases in proportion to the number of processing units assigned to it. For example, if we simulate neuronal activity under a very low spiking rate regime, neurons can be updated almost independently. Therefore, we can increase the size of the system we can handle by simply adding processors, each handling about the same number of neurons, that is, keeping the load on each processor approximately constant and without incurring an overwhelming communication burden.

The purpose of this paper is to determine whether simulating the activity of a large-scale neuronal network can be achieved in a practical amount of time, even when the neuronal model contains a very high level of detail due to the specific requirements of epilepsy research. If so, what strategies could make this possible? How flexible will the resulting model be in terms of being capable of testing different biophysical models and thus understanding which factors determine their emergent behavior? Will they be efficient enough to allow us to explore, at least in part, the parameter space and thus generate hypotheses that can be tested experimentally? Our simulations are based on parallel programs developed in our laboratories [6, 21, 24–27], but we will make reference to other large-scale work [7] and related research even when it does not cover the network sizes we are interested in [4, 5]. When comparing our previous work with these models, this paper specifically addresses how computer architecture and software design affect parallel performance, particularly in terms of how the parallelization is implemented and the various simultaneous calculations are coordinated.

## 2. Materials and Methods

### 2.1. From Neurons to Networks

To understand massive network phenomena such as epileptiform activity, it is necessary to model sufficient numbers of neurons and to model them in sufficient detail. Moreover, it is necessary to run models that vary both in complexity and nature in order to generate meaningful new hypotheses. Indeed, efficient large-scale detailed models of neuronal activity have recently become relevant to develop understanding because they can help resolve the following.

- Questions that are meaningful biologically: experimental setups are starting to produce data that could be compared with these models quantitatively because of both the microscopic details and large-scale data, for example, multichannel recording in implanted arrays, such as electrocorticography (ECoG), electroencephalogram (EEG), calcium imaging [28], and in vitro multielectrode arrays (MEAs).
- Questions that are meaningful computationally: computational tools such as supercomputers have evolved to the point where simulations of a realistic detail and size appear to be manageable (e.g., [7]).

To add to the complexity of these modeling approaches and their numerical demands, the necessity of performing validation runs has to be considered in order to determine the sensitivity and the statistical properties of these nonlinear models with regard to model parameters, including network geometry.

In the following, we will provide a brief description of how neurons and networks are modeled in our programs; we will provide a single description of the model unless implementations differ.

### 2.2. Realistic Single Neuron Models

We evaluated two simulators, pNeo [25] and Verdandi [27], modeling the microcircuitry of a neocortical area using six distinct cell types, differentiated by morphology and compartment parameters: two excitatory (deep and superficial pyramidal cells) and four inhibitory (three types of basket cells and the chandelier cells). All cells are modeled using a number of cylindrical compartments in order to include spatial effects and extracellular currents.

Superficial and deep pyramidal cells are modeled with five and seven compartments, respectively; the inhibitory cell types are modeled with two compartments (Table 1). pNeo places neurons on a regular grid at a distance of 5 *μ*m from each other for the pyramidal cell types and 15 *μ*m for the chandelier and basket cells. To compensate for this regularity, conduction velocities are partially randomized. In Verdandi, neurons are set on the same lattice as pNeo but have a small random displacement from it. Basic capacitance, conductivity, and channel characteristics are identical for the two models. The interested reader can find a complete and detailed description of those models in our previous work (e.g., [6, 26]).

Compared to other large-scale models [7], our simulations include more cell types, covering the entire depth of the neocortex. While our more complete approach is not necessary to investigate all cortical models, it must be included to realistically model epileptiform activity since it is not known which factors determine its behavior.

### 2.3. Modeling Networks

We simulated rectangular cortical areas with sizes depending on the number of neurons (cell density was kept constant). Network wiring in our model was based on literature data on mammalian neocortex and included specific functional connection types between cell classes, randomized but representative density distributions for each connection class, and both fast and slow connections (e.g., [26]). Long-range cortical connections are included in pNeo for interneural distances of more than 1 mm (networks larger than about 100 K neurons). Excitatory synaptic signals were modeled with an alpha function (time constant 1–3 ms) and inhibitory signals were modeled with a dual exponential function (time constants 1–7 ms). Each of the six groups of cells has its own set of multiplicative tuning parameters to modulate the strength of its connection from other neurons, for either inhibitory or excitatory synapses. These values were kept constant over all pNeo simulations to keep the basic biology of the network invariable, while they were changed with network size for Verdandi in order to keep the network activity similar among different sizes. Detailed descriptions of the characteristics of the networks can be found in our previous work [6, 21, 24–27].

Our simulations do not include columnar structures as many other large and small-scale models do because their existence as anatomic or functional units remains controversial at least to some degree [7, 29]. Moreover, it is unclear how connectivity affects epileptic seizures, which places great value on the ability of a model to explore a more general set of network topologies. In the simulations described here, the probability of having a connection between two neurons is simply dictated by their cell type, location, and distance as described in Visser et al. [27] and the references therein. The absence of columnar structures leads to a much higher connectivity in our models, as will be reported in more detail in the results section.

### 2.4. Designing for Scalable Performance

The two simulators we evaluated in this work, pNeo and Verdandi, included extensive instrumentation for collecting detailed performance data on all aspects of their execution, interprocess communication, and memory usage so that we could evaluate their scaling over a wide range of network sizes on different computing architectures. pNeo was derived from an earlier simulation using p-Genesis ([30]; http://www.genesis-sim.org/project/pgenesis) by removing all overhead codes. Verdandi was created as a more general simulation tool. Both pNeo and Verdandi port easily to most parallel-computing platforms: they need only MPI ([31, 32]; http://www.mcs.anl.gov/research/projects/mpi) and C++. Verdandi can also work with OpenMP ([33]; http://OpenMP.org/wp) to support lighter weight parallelism on multicore nodes and benefit from shared memory access within that MPI process.

The I/O is usually done asynchronously by MPI process: each writes to its own output and log files; input is also read asynchronously. OpenMP threads belonging to the same MPI process on the other hand are synchronized. The MPI environment is needed only to propagate spikes among neurons that belong to different MPI processes. Synchronization was achieved using barriers. When a barrier is inserted into a program, no OpenMP thread (or MPI process, depending on the type of barrier) can proceed past it unless all threads have reached it. This, for example, guarantees that all spikes have been sent and received before moving to the next time step. However, barriers often cause inefficiency, as shown below.

The programs can be downloaded from our laboratory's website (http://epilepsylab.uchicago.edu/page/neuroscience-links).

*pNeo* assigns each neuron to an MPI process based on its spatial location. From there, the program proceeds in parallel: each MPI process determines the characteristics of its neurons and the connections within and across processes are established, after which each process starts the time propagation of its set of neurons. After each node completes its propagation time step, the spike exchange step is performed in a nonblocking fashion by each process independently. All processes wait at a barrier associated with the end of the spike exchange to synchronize before the next time step.

*Verdandi* consists of 3 related packages: *netgen*, which generates networks with specified characteristics (e.g., size and type of neurons); *distnet*, which distributes netgen's cell and network data over the desired number of MPI processes; *sim*, which runs the dynamics. The OpenMP-enabled part of the code covers only the time evolution of the neurons on the nodes for each time step; the remaining operations, principally involved in exchange and handling of node-to-node spike propagation, are done using one thread per MPI process. After the OpenMP threads have completed their independent tasks, an MPI barrier is set, forcing synchronization of all processes to exchange spikes. Further barriers are used to separate the phases for collecting the spikes, pushing them to all processes and connecting each spike event to the local neurons that it targets. It is important to realize that all those barriers produce inefficiencies in the code parallelization because they force a number of computing cores to stay idle while other processes are completed. These inefficiencies do not affect how the overall MPI scaling behaves if they are only slightly affected by the number of MPI processes utilized (see Table 2 and related text). Perfect scaling does not mean perfectly efficient code, just as perfect MPI scaling does not imply perfect OpenMP scaling.

### 2.5. Simulation Approach

We ran our experiments on two distinct large parallel architectures to give us an idea of how generalizable our scaling results are. *Beagle* is a Cray XE6 massively parallel supercomputer with more than 700 shared memory nodes with 32 GB of RAM each. Each shared memory node is made of 4 six-core dies (packaged in two 2.1 GHz AMD Magni-Cours Opterons) for a total of 24 cores per node. It is based on the Cray Gemini interconnect with a folded 4D-torus topology. The latency is expected to be slightly over 1 *μ*s, depending on the operation, and to depend only weakly on internode distance. Gemini is capable of supporting a minimum of 4.7 GB/s bandwidth per direction (a “flattened” 4D-torus is linked in 6 directions: X, -X, Y, -Y, Z, and -Z). For more detail, see https://beagle.ci.uchicago.edu/; http://www.cray.com/Products/XE/CrayXE6System.aspx.

*Fusion* (http://www.lcrc.anl.gov) is a Linux cluster with 320 nodes each with 36 GB of RAM (16 have 96 GB of RAM). Each shared memory node has a dual 2.53 GHz 4-core Intel Nehalem Xeon for a total of 8 cores per node. The cluster is based on an Infiniband QDR interconnect with a flat tree topology (i.e., all nodes are connected to the same switch). The latency is expected to be around 2 *μ*s. Bandwidth should be 4 GB/s per link. Therefore, the most obvious differences between the two machines are processors (2.1 versus 2.53 GHz or a 83% difference in clock rate), cores per node (24 versus 8, therefore a considerable difference in number of cores, memory per core, and messaging conflicts), bandwidth, and latency (potentially affecting scaling).

Verdandi is able to take advantage of both distributed-memory parallelism using MPI processes and shared memory parallelism within a multicore node using threads. This allows us to gain some understanding in the performance tradeoffs of replacing MPI processes with threads.

To simplify the interpretation of the results, we made the runs as similar as possible across both sizes and machines. Therefore, we used combinations of MPI processes that respected the symmetry of both machine's nodes (i.e., we used 4 MPI processes per node, and thus not all cores could be utilized in most of our simulations).

We simulated networks of 8.8 K, 35 K, 98 K, and 390 K cells with pNeo and of 2.6 K, 32 K, 94 K, and 380 K for Verdandi. The differences in network sizes between pNeo and Verdandi runs are incidental and immaterial to the scaling results presented here.

The number of MPI processes considered in the scaling simulations ranged between 4 and 256. Not all combinations of MPI processes and network sizes were considered: on few cores, large networks require too much memory per process or take too much time to be practical; on many cores, running small networks leaves each process with too few neurons to efficiently amortize interprocess communications. Large processor pools were not considered because we did not want to simulate networks that would be too large to be realistic in our understanding of epilepsy given the modeling approaches followed by pNeo and Verdandi. More precise modeling of the longer range connections would be required for such runs to be biologically meaningful.

To start activity at the simulation onset, a subset of cells received a current injection. The system was propagated for a total of 0.4 and 0.5 seconds for Pneo and Verdandi, respectively. Verdandi computation times here include only network upload and dynamics propagation; that is, they do not include network generation. 

## 3. Results and Discussion

### 3.1. Biophysical Behavior

**Figure 1:**
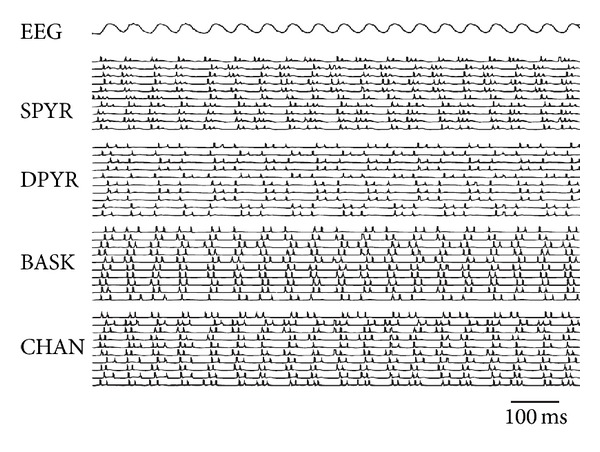
Example of emergent behavior produced by this type of simulation (using Verdandi). Seizure-like oscillation in a patch of 90 K neurons as displayed by the pseudo-EEG. Below are the action potential trains of ten representative cells of each kind: superficial pyramidal cells (SPYR), deep pyramidal cells (DPYR), large basket cells (BASK), and chandelier cells (CHAN). The ten cells are selected from different locations in the simulated network.

The goal of this type of model is to study the relationships between cellular and population activity.  Figure 1 shows part of a typical result of a 90 K simulation with an oscillatory pseudo-EEG and a representative selection of associated cellular activities. Note how each of the single cell behaviors corresponds to the EEG trace, which is impossible to do experimentally for 90 K cells.

### 3.2. Memory Scaling

The expected relationship between network size and required memory is straightforward. If the simulated cortex volume is relatively small (small *N*, number of neurons), the total number of connections in the cortical area and thus the memory occupation of the interneuron connection data will be quadratic: ~*probability  of  connection *×*N* × *N*. As the volume increases (*N* larger), the fraction of neurons to which each neuron is connected becomes smaller because the probability of having a connection is distance dependent and subject to a cutoff. This cutoff ranged between 100 and 900 *μ*m depending on the type of connection, with the most frequently occurring excitatory-excitatory ones at 500 *μ*m. Eventually, increasing the total number of neurons will have no effect on the number of connections for each of them and memory occupation will become linear in *N*, *~probability  of  connection *×*N*_max⁡_ × *N*.  In the regime simulated here, the storing of inter-neuron connection data should approximate *c*_1_(*N*)*N* ^*c*_2_(*N*)^, where  *c*_1_(*N*) is equal to  *k*  for small networks and *kN*_max⁡_ for very large networks and 1 < *c*_2_(*N*) < 2. Thus, it should produce simple plots on a log-log scale. On the other hand, the information about the status of neurons scales as  *c*_3_ *N*, with  *c*_1_(*N*) < *c*_3_  for small networks and  *c*_1_(*N*) ≫ *c*_3_  for very large ones [4]. Therefore, for networks of at least a few thousand neurons, we expect memory use to be dominated by connectivity.

**Figure 2:**
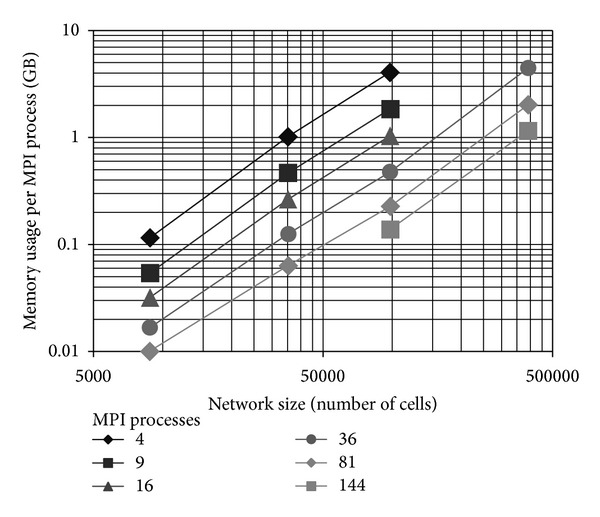
Maximum memory usage per process for pNeo on Beagle as a function of network size on a log-log plot with basis 10. Different lines depict simulations that used a different number of MPI processes. Results on Fusion were essentially identical. The results for Verdandi were qualitatively similar. The total memory used by each simulation can be obtained by multiplying these numbers by the number of MPI processes and it is reasonably constant for each network size.

In pNeo, the memory needed per core for each network size was reduced as the number of MPI processes used was increased keeping the total approximately constant (see Figure 2). However, for larger numbers of MPI processes duplications eventually become important; for example, for the smallest network, running the computations on 9 and 144 cores used a total of 0.48 GB and 0.86 GB, respectively. Verdandi's behavior was similar except that the small network-size region was dominated by fixed memory usage (~100 MB per core). In the region around 100 K cells, the two programs had very similar memory use, around 20 GB in total. For larger networks, the total number of connections for each network simulated became almost linear in Verdandi while it kept growing superlinearly in pNeo, consistently with the latter including long-range interactions. (In Verdandi, the total number of connections for each network size was 0.5 M for 2.6 K, 31 M for 32 K, 0.13 G for 94 K, and 0.64 G for 380 K, while in pNeo had 4.2 M for 8.8 K, 38 M for 35 K, 0.16 G for 98 K, and 1.6 G for 390 K.) In the following analysis, pNeo will be used in order to produce estimates that are both more realistic (as it includes long-range interactions which can affect memory considerably for large networks) and more conservative. We based our estimates on the smallest MPI pool that spanned a realistic memory use, 9-MPI processes, and the largest used, 144-MPI processes, to reduce artifacts. Data were fit with a simple regression based on log-transformed data, using the function lm of R (http://www.r-project.org/). We used a relatively simple model because the plots are very close to straight lines (confirmed by the *r*-squared values) and to avoid overfitting:  *Memory* = *α* · *N* ^*β*^, where *Memory* is in GB. We estimated *α* = 0.5*E* − 7 GB and  *β* = 1.5 with an adjusted *r*-squared of 0.998 (to confirm the results, the 144-MPI process simulation had  *β* = 1.4 and  *α* = 2.5*E* − 06 GB with all the points and  *α* = 1.3*E* − 06 GB excluding the smallest network—the variability in the value of  *α*  is relatively unimportant for this extrapolation). Using these estimates, the amount of RAM necessary to simulate 1 and 10 million cell networks, representative for generating the aggregate signal picked up by a single EEG electrode, would be 760 GB and 26 TB, that is, within the capability of modern supercomputers. It is important to realize that even a small increase in connectivity would affect these estimates drastically, for example, if we set  *β*  to 1.6 or 1.8 the estimates for a 1 million cell network become 2 and 31 TB, respectively.

### 3.3. Execution-Time Scaling

**Figure 3:**
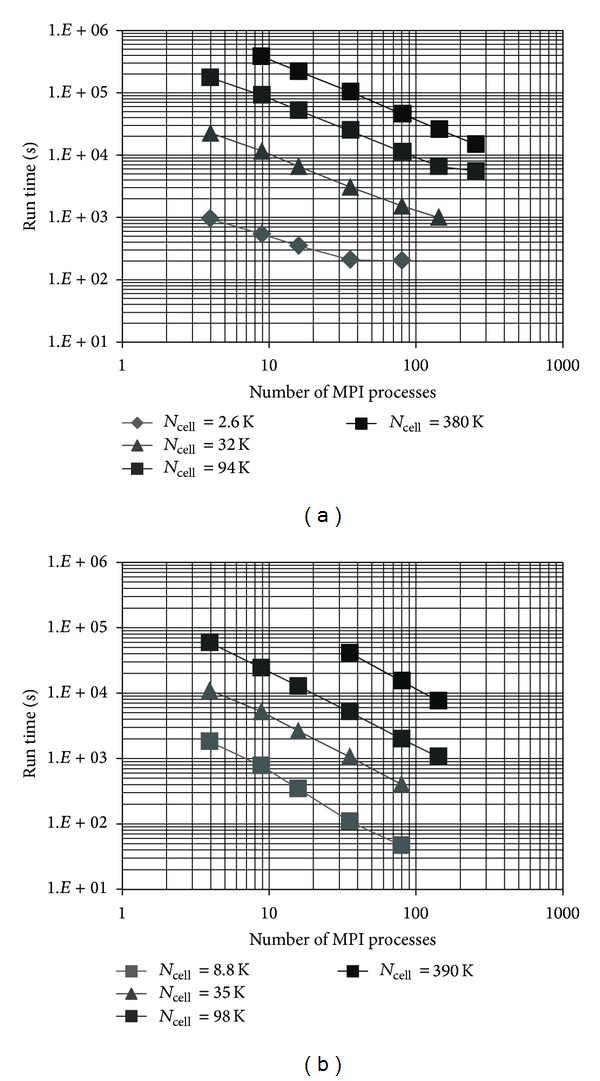
Strong scaling plots for calculations performed on Beagle: (a) Verdandi and (b) pNeo. Run time is the time it took to complete a simulation, from beginning to end. Most of the measurements exhibit nearly perfect strong scaling.

Our programs exhibited near perfect strong scaling as the number of MPI processes used increased (Figure 3). The main deviation from linear scaling was found for Verdandi when using large MPI-process pools, which suggests that synchronization and communication issues started to become more important (e.g., rightmost points in Figure 3(a)). This is consistent with local calculations becoming much smaller and faster and needing to communicate with more and further away nodes, thus needing to synchronize at barriers a lot more often. Indeed the time spent synchronizing increases with MPI process pool size (Table 2). The time spent on barriers increases most dramatically for the smallest network and it is consistent with the strong scaling plot becoming almost flat near the end (i.e., no gain from parallelization): this small network simply cannot be run efficiently on such a large machine. Excluding the latter network size, the time spent on barriers increases with network size most likely because of changes in activity levels, thus affecting both the time evolution of the single neurons and the number of spikes that need to be transmitted. Excluding the 4 MPI-process data point (which was run entirely on a single node and therefore did not use the interconnect), the time spent on barriers changes only around 10~15% when more MPI-processes are utilized and therefore did not affect the scaling appreciably.

Therefore, in the case of Verdandi it is possible that synchronization issues will affect weak scalability even more seriously for future models, which will have higher connectivity.

On the other hand, pNeo scaled very well for all network sizes and MPI pools in our test set. From the plots it appears that pNeo is two to three times faster than Verdandi for the same problem size even without considering threads (otherwise it would be about 20 times faster) or the larger number of connections included in pNeo for larger network sizes.

In Table 3 we try to extrapolate the weak scaling properties of the two programs. We are not presenting weak scaling in a conventional way because keeping the load per processor constant would require quantities difficult to interpret (e.g., network size at some variable power). Instead, we studied how computation time changed when we kept the number of neurons per node nearly constant. Since keeping the number of neurons per node constant would have been impractical in our simulations, we estimated some of the values in the table from the strong scaling results (Figure 3, and this should have a negligible effect since it essentially requires interpolation—extrapolation for the largest network size—on almost straight lines: *r*-squared > 0.99). Verdandi shows a flattening of the expected computation time for larger sizes—at least in part because the average number of synapses per neuron is becoming constant. Keeping the total time required about the same as for the larger simulations, of the order of two to three hours, we would need 1.5 K and 15 K MPI processes to simulate one million and ten millions neurons, respectively. pNeo's expected computation time is still increasing even if with a flattening slope. Extrapolating from Table 3, pNeo would require 450 MPI processes for one million neurons and about 4.5 K for ten million. If we assume that the time will grow linearly from the last two points, a fairly conservative assumption, the time required for the one and ten million neuron patches would be approximately 4 and 25 hours, respectively, that is, both simulations could be done easily on any supercomputer.

The results obtained on Fusion showed the same behavior, with very good strong scaling plots and very predictable behavior. For Verdandi, to understand the different performance on the two architectures (about 4 times faster on Fusion on a per core basis), the effect of using a shared memory approach on computation time needs to be considered.

### 3.4. Behavior of Mixed Simulations

Shared memory parallelism can circumvent interprocess communication overhead. We measured the tradeoff in performance by exchanging MPI process-level parallelism against OpenMP thread-level parallelism. Verdandi can make very effective use of shared memory: for the simulations we tested changing from 1 to 24 threads increased the total memory used by only 25%.

As for the MPI processes, it is expected that the total time will increase slightly when very good scaling is observed (producing the approximately 1/*n* decrease in wall time observed previously). Multithreading becomes useless when the total time increases proportionally to the number of threads.

**Figure 4:**
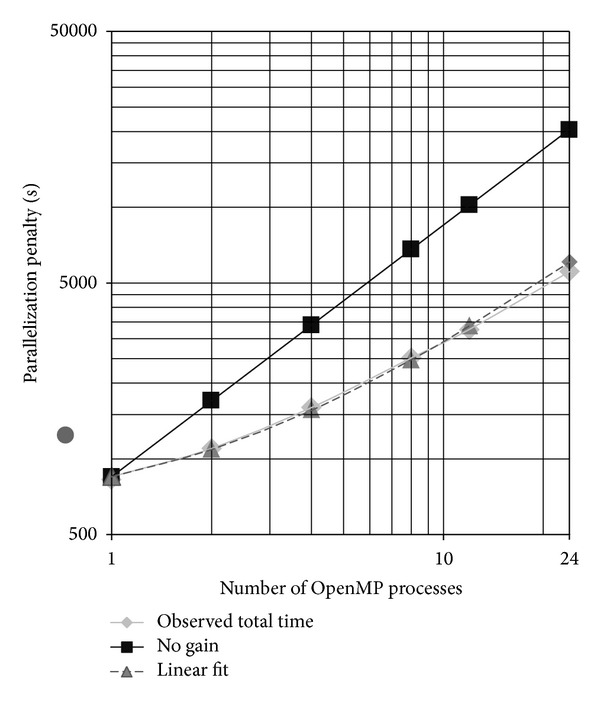
Increase in total time (sum of the time taken by each thread in a simulation and called “Parallelization penalty” because it is the total CPU time allocated to the simulation) as the number of OpenMP threads is increased while keeping the number of MPI processes at 1 (single node). This calculation was performed on Beagle.

Figure 4 displays the total computation-time as a function of the number of OpenMP threads for a single node calculation. The fit to a linear model, also plotted in the figure, is very good, indicating that a simple model might explain this behavior. Overall, OpenMP produced a speedup of a little less than 4 for this calculation when using 24 cores and a little more than 2 when using 6.

Therefore, while using OpenMP reduced the wall clock time required to complete a task, the maximum speedup was 6 times less than expected when using 24 MPI processes. There can be multiple reasons for this behavior, which is usually caused by serial calculations and/or conflicts in the use of fast memory (cache) and other resources [23]. In this case, the good fit we have with a linear model suggests that there is a sizeable serial part in the calculations, consistent with Verdandi's implementation and Amdahl's law [34], with about a 1/3 of the single thread time becoming serial, thus increasing the total time linearly in the number of cores because they would all sit idly, plus a 2/3 parallel part whose total time would be unaffected by the number of cores. Indeed, the profiling runs show that the time spent waiting on barriers (meaning threads halted waiting for some process to complete) increased from 0 to 25, 30, 60, and 75% as the number of threads increased from 1 to 2, 4, 8, and 12 (note that this has no effect on Figure 3 since the number of OpenMP threads per MPI process is kept constant). Different approaches for scheduling threads (OpenMP schedule set to “dynamic,” “guided,” or “static” [33]) failed to produce meaningful differences in performance, confirming that time is spent on barriers largely because of serial processing.

**Figure 5:**
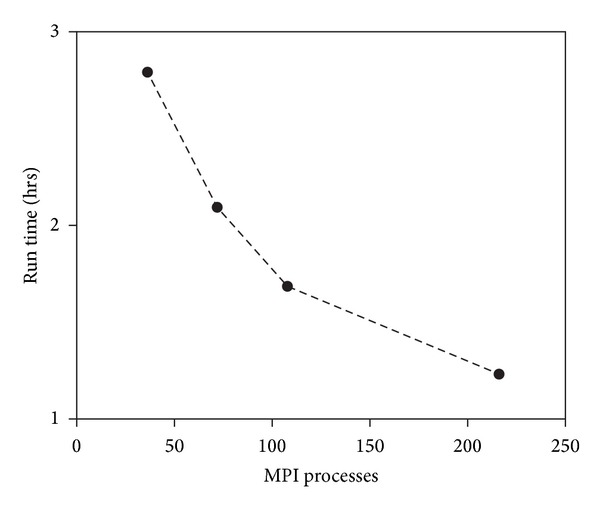
Run time (wall clock time here, not total time as in the previous plot) for a 100 K-cell network on 9 nodes (216 cores) of Beagle. The number of MPI processes was varied while the number of OpenMP threads was adjusted so that each run used all 216 available cores. The leftmost point corresponds to 3 MPI processes per node with 8 OpenMP threads per process.

In Figure 5, the tradeoff between MPI processes and OpenMP-threads is shown. We have seen that halving the number of MPI processes should double the CPU time (Figure 3), while doubling the number of the threads should reduce it by 1.5 (Figure 4). Indeed, the second point from the right in Figure 5 indicates a factor of 1.33 times larger than the point to its right. As more MPI processes are replaced with threads, Figure 5 shows better scaling than that shown in Figure 4, most likely because the simulation ran longer, reducing the effect of I/O, which is not parallelized.

Now, let us reconsider Verdandi's performance difference in completing the same simulation as a hybrid calculation (MPI + OpenMP) on Beagle or as a pure MPI calculation on Fusion. For example, on Beagle it took a total of about 5 K seconds to simulate the dynamics of 300 K neurons, while it took only about 1.3 K seconds on Fusion. On Beagle there were 6 OpenMP threads for each MPI process. From the OpenMP scaling results we expect the total time to increase by a factor of 4 when 6 threads are used to replace 6 MPI processes, 1.3(Ks) × 4≅5(Ks), which explains most of the performance difference.

### 3.5. Discussion

We showed that our complex and highly connected neuronal network models can exhibit nearly perfect linear scaling for biologically meaningful parameter values. Even if, as the network is distributed over more and more nodes, communication should eventually dominate other aspects and limit the scalability of the models, this did not appear to become a serious issue within the scenarios we presented here, at least for pNeo. Verdandi spent a considerable amount of time on barriers, both for MPI (synchronizing communication) and OpenMP (mostly waiting for serial parts and synchronizing threads): the combined effect could easily put the total amount of idle CPU time over 90% of the total used for computation. We believe that this fraction can be reduced considerably by using approaches more similar to pNeo (e.g., removing barriers, using nonblocking messaging and one-sided communication).

The memory utilization in large-scale simulations comparable to ours [7] was 2.8 TB for a 22 M neuron simulation having 11 B synapses (about half of the connections expected from Verdandi for a network of this size, as Verdandi is already in a linear regime at 100 K, and much less than the number of connections expected in a pNeo simulation of that size). In the same paper, [7], a 2 M neuron 2 G connection simulation took about 20 minutes to run 1s of simulation on 4 K processors. The computation time is of about the same magnitude as pNeo and the difference is in line with the higher connectivity of our model (pNeo has about 2G connections already for a 400 K network) and the extrapolative nature of our large-network estimate. Therefore, their finding is in line with our projections given the differences in connectivity between our models.

Model complexity is still the main limitation of our models: we believe that to have an accurate understanding of epileptiform activity it is necessary to at least include gap-junctions and possibly plasticity and other types of neurons or an even higher level of detail in the current model types. Moreover, Verdandi did not include long-range interactions. However, the purpose of this work was to evaluate current tools and develop an understanding of whether more complex models could be run with current simulation tools, which is quite relevant to current trends in neuroscience research [18, 19]. The lack of gap junctions is unlikely to affect our estimates for memory scaling for more complex models since they are local in nature; however, they are likely to affect our time extrapolation. We believe that the conservative nature of our extrapolations should be sufficient to account for their effect. Implementation of plasticity rules requires monitoring of activities and implementation of a rule to adjust synaptic coupling strength between the neurons. Therefore, we do not expect it to affect the scaling sufficiently to alter our projections enough to change our conclusions. Another potential limitation is that we did not explore larger networks and/or processor pool sizes. As explained previously, we do not believe that this would have been a good use of resources because the models are still too simple to represent epilepsy faithfully at larger scales, which implies that the results would not have practical scientific value. Moreover, we believe that computation time and memory use behaved very predictably rendering the information provided by such simulations of limited computational value.

## 4. Conclusions

Both MPI processes and OpenMP threads allowed faster simulations; however, these results suggest that our current implementations produce more gains with the former, which should therefore be preferred when possible. Our results also indicate that for large-scale neuronal network simulations, shared memory parallelism with OpenMP can provide an efficient alternative to MPI process-per-core as long as the most time consuming phases of the computation are implemented to take advantage of it. However, it is crucial to very carefully implement synchronization, IO, and barriers.

The object-oriented approach used in Verdandi allows for more straightforward and faster testing of alternative approaches in terms of networks and neuronal models. However, this is associated with computational cost for this specific implementation. In general, it is not possible to create this type of flexible model without paying a performance penalty, but we made this choice because the advantage it gives in terms of flexibility and reliability outweigh the computational overhead, given the complexity of neuronal network simulations.

## Figures and Tables

**Table 1:** Size (diameter and length) of cells compartments in *µ*m [26].

	Soma (*µ*m)	D1 (*µ*m)	D2 (*µ*m)	D3 (*µ*m)	D4 (*µ*m)	BD (*µ*m)	IS (*µ*m)
SPYR	16.1, 22	2.0, 140	3.3, 190	—	—	2.4, 200	2.2, 50
DPYR	16.1, 22	2.0, 250	2.9, 400	4.4, 400	4.7, 400	6.3, 200	2.2, 50
BASK1	16.1, 22	2.0, 900	—	—	—	—	—
BASK2	8.0, 11	2.0, 600	—	—	—	—	—
BASK3	5.4, 5.5	2.0, 300	—	—	—	—	—
CHAN	4.0, 5.5	2.0, 150	—	—	—	—	—

Rows contain cell types (SPYR: superficial pyramidal, DPYR: deep pyramidal,  BASK1,…, BASK3: basket cells of type 1 to 3, CHAN: chandelier cell) while columns represent the various segments (Soma, D1,…, D4: dendritic compartments 1 to 4, BD: basal dendrite, IS: axon initial segment).

**Table 2:** Percentage of time spent at an MPI barrier as a function of the network size and pool of MPI processes used for Verdandi.

Network size	Number of MPI processes used					
	4	9	16	36	81	144
2.6 K	16%	34%	42%	56%	77%	N/A
32 K	12%	25%	27%	34%	43%	53%
94 K	21%	35%	38%	42%	46%	51%
380 K	N/A	44%	48%	53%	56%	57%

**Table 3:** Estimated values for weak scaling problem for Verdandi (first block) and pNeo (second block).

Network size	Closest actual MPI pool from simulation	Estimated size of MPI pool	Expected computation Time (s)
Verdandi			
2.6 K	4	4	0.94 K
32 K	36	49	2.2 K
94 K	81	144	6.2 K
380 K	256	576	6.6 K

pNeo			
8.8 K	4	4	1.8 K
35 K	16	16	2.6 K
98 K	36	45	4.1 K
390 K	81	178	6.8 K

“Closest actual simulation” is an MPI pool that was actually run for that network size. “Estimated size of MPI pool” is the number of processes necessary to keep the ratio of number of cells/number of processors constant. The estimate time is computed assuming the observed scaling laws either with interpolation (up to 94 K for Verdandi, and up to 98 K for pNeo) or with extrapolation on a straight line (the larger network).
